# Extra-articular manifestations of rheumatoid arthritis: a hospital-based study

**DOI:** 10.4103/0256-4947.51774

**Published:** 2009

**Authors:** Aisha Al-Ghamdi, Suzan M. Attar

**Affiliations:** a From the Department of Internal Medicine, King Abdulaziz University Hospital, Jeddah, Saudi Arabia; b From the Department of Rheumatology, King Abdulaziz University Hospital, Jeddah, Saudi Arabia

## Abstract

**BACKGROUND AND OBJECTIVE:**

The frequency of extra-articular manifestations in rheumatoid arthritis (ExRA) differs from one country to another, so we investigated ExRA frequency in a well-defined hospital patient population with rheumatoid arthritis (RA) in Saudi Arabia. We also examined possible predictors of the development ExRA.

**METHODS:**

A retrospective analysis was conducted of all patients diagnosed with RA at a university hospital during a 4-year period. Cases were classified according to the 1987 American College of Rheumatology criteria for RA, and the frequency of ExRA was recorded.

**RESULTS:**

Of 140 patients who fulfilled the criteria for the diagnosis of RA, 98 (70%) developed ExRA features. Anemia occurred in 61%, thrombocytosis in 16%, pulmonary involvement in 10%, and renal amyloidosis, vasculitis and Felty syndrome were present in 6%, 2% and 1%, respectively. The mortality rate was high (16%) in patients with ExRA. The predictors for mortality were lung involvement, age over 50 years and kidney amyloidosis.

**CONCLUSION:**

ExRA were present in a substantial proportion of our patients, which lead to a worse disease outcome. Anemia, thrombocytosis and respiratory system involvement were the commonest. Early recognition and treatment are important to decrease mortality.

Rheumatoid arthritis (RA) is a chronic autoimmune disease characterized by inflammatory polyarthritis and systemic features, with a prevalence of 1% in Western populations.^1^ In one study in Saudi Arabia, the prevalence was 0.02% in the Al-Qassem region.^2^ Extra-articular manifestations of rheumatoid arthritis (ExRA) occur in about 40% of patients, either in the beginning or during the course of their disease.^3^ The presence of ExRA is associated with severe active disease and increased mortality compared to the general population.^4^,^5^

The frequency of ExRA specific manifestations is highly variable because of case definitions and study design. For example, nodules are easily identifiable and their incidence was reported as 30%, while pulmonary fibrosis is not clinically apparent and has a reported incidence in population studies of 6.1% compared with 30% in case findings.^6^ Using previously applied definitions for ExRA is of major importance to unify the diagnosis.^7^,^8^

There are no reliable predictors for the development of ExRA, although many have been suggested.^7^–^9^ They include such constitutional factors as male sex, association with HLA-related shared epitope genes, autoantibodies such as rheumatoid factor (RF), antinuclear antibodies (ANA) and anti-cyclic citrullinated peptide antibody (anti-CCP), as well as environmental factors such as smoking.^10^–^15^ Due to the systemic inflammatory process, the incidence of mortality in RA is increased by four-fold compared with the general population.^16^ Although few patients die of the disease, or of specific complications such as cervical instability or drug side effects, the major factors that predict mortality are the presence of co-existing heart and lung disease, malignancy and dementia.^17^ Other suggested factors that predict mortality are active disease despite medical treatment and the presence of ExRA.^18^ In this study, we reviewed the incidence of ExRA retrospectively at a university hospital in Jeddah, Saudi Arabia, and identified clinical and biochemical risk factors for developing ExRA.

## METHODS

This retrospective study was conducted at King Abdulaziz University Hospital, the only teaching governmental hospital in Jeddah. The facility provides health care to a multinational population of mixed socioeconomic status. A computerized retrieval system was used to identify all patients with a registered diagnosis of RA who received treatment at the hospital during a 4-year period (1 January 2004 to 30 December 2007). RA patients were identified according to the 1987 American College of Rheumatology classification criteria for RA (the most current criteria for RA diagnosis).^19^ The data collected from records included demographic features, clinical findings, including duration of the disease at the time of the study (classified as <1 year, ≥1 to 5 years, and >5 to 10 years, and >10 years), disease activity (based on the number of tender/swollen joints or the disease activity score obtained from the 28-joint count),^20^ treatment, the presence of articular and extra-articular manifestations of RA, a positive family history of RA and associated co-morbidities, laboratory parameters (complete blood count, rheumatoid factor [RF] and antinuclear antibody [ANA]), and radiological changes (characteristics of lesions on chest radiographs and the typical radiological features of involved joints).

Extraarticular features included renal invovlement defined as urinary excretion of more than 500 mg protein/24 hours, cellular casts not attributable to infection, or abnormal histology on renal biopsy. Amyloidosis was considered if confirmed by biopsy or fine needle aspiration. Respiratory involvement was defined as pleuritis or interstitial lung disease, scarring and the formation of nodules in the lungs documented by a high-resolution CT scan of the chest. Pericardial involvement was considered if pericarditis and/or pericardial effusion was documented in the clinical record as related to RA. Nervous system involvement was considered compression neuropathy (e.g. carpal tunnel syndrome) and peripheral neuropathy. Sjogren syndrome was defined by dryness of the eyes (xerophthalmia) and mucous membrane (xerostomia). Rheumatoid nodules were defined as subcutaneous nodules with a diameter ≥5 mm in the extensor surfaces of the extremities and fingers. Hematological changes included leucopenia (WBC count <4×10^9^/L), thrombocytosis (platelet count >400×10^9^/L), anemia (normochromic, hypochromic or megaloblastic). Felty syndrome was considered present if there were neutropenia plus splenomegaly documented by ultrasound in the absence of other causes in the RA patient. Rheumatoid vasculitis was defined as the presence of mononeuritis multiplex or acute peripheral neuropathy, peripheral gangrene, histological evidence of necrotizing arteritis, or deep cutaneous ulcers. Ischemic heart disease, lung disease, infection and malignancy were considered co-morbid illnesses. Gender, duration of the disease, RF, ANA and smoking (either active or had stopped within the last 5 years) were evaluated as risk factors for the development of ExRA. We examined the effect of age, gender, RF and the presence of co-morbid illness on the mortality rate in patients with ExRA.

Data analysis was done using Statistical Package for Social Sciences (SPSS software, version 11). Mean and standard deviation were calculated for quantitative data and proportions for categorical variables. One-Way ANOVA and the t test were used for comparing means of continuous variables. Proportions were compared by the chi-square test and the Mantel-Haenszel test as required. Results were considered significant if the *P* value was less than.05. Multivariate logistic analysis was used to evaluate possible predictors of mortality.

## RESULTS

One hundred ninety-two patients were diagnosed as having RA. Fifty-two were excluded due to incorrect diagnosis (such as registration error, premature diagnosis, or the case records did not contain enough data to support the diagnosis). One hundred forty fulfilled the 1987 American College of Rheumatology classification criteria for the diagnosis of RA. A total of 98 of 140 (70%) had features of ExRA.

Seventy-four patients (75%) were women while 24 (25%) were men. Their mean (SD) age at the time of the study was 47.2 (15.6) years for females and 44.6 (13.2) for males. Sixty-one percent patients (62%) were Saudi. Duration of the disease at the time of the study is illustrated in Table 1. Three percent of our patients were smokers. Only two patients had a family history positive for RA. The main extra-articular features are shown in Table 2. Anemia was found in 60 patients (61%). Iron deficiency anemia was the commonest, affecting 52 of 60 anemic patients (87%). Normochromic normocytic anemia affected 8 patients (13%). Thrombocytosis, whether considered reactive to iron deficiency anemia or as an RA activity marker was found in 16 patients (16%). Respiratory system involvement was a common feature, affecting 10 patients (10%) in the form of interstitial lung disease and pleuritis in 7 patients (7%), while leukopenia was found in only 5 patients (5%). The lymph nodes were enlarged in 4 patients (4%). Carpal tunnel syndrome and rheumatoid nodules were present in 3 patients (3%) while Sjogren syndrome and leg ulcer were present in 2 patients (2%). Pericardial effusion and Felty syndrome were the least frequent feature found in RA patients, occurring in 1 patient only (1%). Pericardial effusion was detected in 1 patient (1%). Vasculitis was found in 2 patients (2%).

**Table 1:** Duration of the disease at the time of first presentation.

Number of years	Number of patients (n=98) n (%)	Sex (M:F) n (%)	Mean (SD) age in years
<1 year	6 (6)	1:5 (17:83)	38 (15.3)
≥1-5 years	25 (25)	7:18 (28:72)	38.5 (15.3)
>5-10 years	29 (30)	7:22 (24:76)	49.2 (13.3)
>10 years	38 (39)	9:29 (24:76)	51.3 (13.9)

**Table 2:** Main extra-articular manifestations of rheumatoid arthritis.

Extra-articular manifestation	Number of patients (n=98) n (%)
Pericardial effusion	1 (1)
Felty syndrome	1 (1)
Leg ulcer	2 (2)
Sjogren syndrome	2 (2)
Rheumatoid nodules	3 (3)
Carpal tunnel syndrome	3 (3)
Lymphadenopathy	4 (4)
Amyloidosis	6 (6)
Respiratory involvement	10 (10)
Anemia	60 (61)
Normochromic normocytic	8
Iron defeciency	52
Leukopenia	5 (5)
Thrombocytosis	16 (16)

RF was detected as positive in 72 patients (73%), according to our laboratory reference (set point range, 0-20 IU/L). ANA was positive in 37 (38%) patients. Only 20 patients (20%) performed a urine analysis. Proteinuria was found in 10 (10%) and 2 (2%) had RBC casts as well as proteinuria. Renal amyloidosis was observed in 6 patients (6%). Radiological changes were documented as periarticular erosions in 28 patients (29%) and atlantoaxial sublaxation in 4 patients (4%). Thirty-four patients (35%) had the disease in its active form. Fifty-three patients (54%) were receiving treatment in the form of disease-modified antirheumatic medication. Co-morbidities were observed in 25 patients (21%) (Table 3).

**Table 3:** Associated co-morbidities in rheumatoid arthritis patients.

Co-morbidity	Number of patients (n=98) n (%)
Tuberculosis	6 (6)
Hypertension	5 (5)
Diabetes	4 (4)
Malignancy	3 (3)
Coronary artery disease	3 (3)
Cerebral vascular accident	2 (2)
Thrombosis	2 (2)

In RA patients with ExRA, the mortality rate was 16% (16 patients). In the multiple logistic regression analysis, there were certain predictors for mortality among our patients; respiratory system involvement was the major predictor with a significant statistical association (*P*=.001). The second predictor for mortality was the age of patients, as mortality was higher in those aged 50 years or older (*P*=.01). Kidney amyloidosis was the third significant associated factor (*P*=.05) while sex difference and RF did not show any significant association with mortality (*P*=.08, *P*=.9, respectively).

## DISCUSSION

The frequency of ExRA was 70% among our patients. This is higher than in North American populations (40%), but similar to the British population (68%).^1^–^5^ Based on our search of the literature, no previous study has evaluated the percentage of ExRA in the western region of Saudi Arabia. Anemia was the most common ExRA in our population, occurring in 61% of patients, while thrombocytosis occurred in 16%, followed by interstitial lung disease in 10%. Comparing our study to Western reports, iron deficiency anemia was much higher among our patients, which might be explained by chronic blood loss due to drug side effects or poor dietary intake of iron-rich food.^21^ The prevalence of interstitial lung disease was similar to that in previous reports.^6^,^20^ Kidney amyloidosis, found in 6% of patients, is frequently present late in the disease and is related to worse outcomes, including mortality^18^,^21^

Only a small proportion of our patients (20%) had routine evaluation of their kidney function, which is a limitation of the study. The results may have differed if all patients had evaluation of their renal function. We recommend more frequent evaluation of renal involvement in patients with RA, especially in patients with a prolonged disease to evaluate proteinuria and casts. Surprisingly, rheumatoid nodules, which are reported to occur in 30% of patients,^20^ were only found in 3% of our population, which may have been due to underreporting of rheumatoid nodules. Other classic features (secondary Sjogren syndrome, Felty syndrome and neuropathy) were the least common, occurring in 3%, 2%, and 1% of the patients, respectively.

Our study showed that most patients who developed ExRA had positive rheumatoid factor (detected in 73% of our patients) and this was similar to previous reports.^20^ Data are conflicting as to whether the occurrence of ExRA decreases with prolonged disease duration. Turesson et al demonstrated that ExRA does not decrease during a 10-year follow-up.^22^ Our study showed a similar finding, as our patients reported more ExRA with prolonged disease duration (>10 years). Although smoking was suggested as a risk factor for the development of ExRA, we could not relate a significant association in our study as only 3% of our patients were active smokers.

RA is associated with a high risk for morbidity and premature death secondary to the earlier development of cardiovascular, lung diseases and malignancy.^16^–^18^ In our patients, the overall mortality rate was observed to be high (16%), with the following predictors: respiratory system involvement (*P*=.0001), age older than 50 years (*P*=.01) and kidney amyloidosis (*P*=.05). When we compared males with females, sex had little effect on the disease pattern, severity, or the presence of ExRA and mortality.

Our findings may differ from those found in Western patients, which could be due to different human leukocyte antigen (HLA) associations with RA between Caucasian and other populations. RA in the Saudi population is associated with HLA DR10, while in Caucasians it was associated with HLA DR4 and HLA DR1.^23^

Because our study was retrospective, our analysis was limited to case-recorded data collected by the managing physician. On the other hand, the observations documented by the treating doctor reflected manifestations considered clinically relevant. We recommend further prospective studies to document the predictors for the development of the ExRA and outcome in our society. We recommended further research to ascertain the mortality rate in RA patients with and without ExRA and to define the individual risk factors associated with high mortality.

We have demonstrated that ExRA is present in a substantial proportion of a hospital-based sample and is generally associated with a worse disease outcome. Patients with ExRA have a high mortality rate, which could be related to the complication of the disease itself or the presence of other co-morbid illnesses. ExRA needs to be recognized and managed early to decrease the mortality rate.
